# Supplementary material for: Trivalent mRNA vaccine-candidate against seasonal flu with cross-specific humoral immune response
Source: Front Immunol. 2024 Apr 16;15:1381508. doi: 10.3389/fimmu.2024.1381508 (PMC11058219; doi:10.3389/fimmu.2024.1381508)
Supplement: Supplementary file 1 [file DataSheet_1.docx]

Supplementary Material

# Supplementary Data

**Table S1.** Characteristics of mRNA-LNPs after formulation.

| **Name of substance** | **Final volume, mL** | **Size of LNPs, mean with SD, nm** | **PDI, mean with SD** | **Zeta potential, mean with SD, mV** | **[c] mRNA with SD, ng/mkL** | **EI with SD, %** |
| --- | --- | --- | --- | --- | --- | --- |
| H1-mRNA | 4.25 | 69±0.435 | 0.108±0.007 | -10.6±1.39 | 62±5.27 | 89±1.4 |
| H3-mRNA | 3.6 | 71±0.71 | 0.148±0.004 | -8.8±1.5 | 85±1.96 | 88±1.2 |
| BV-mRNA | 4.25 | 68±0.6 | 0.105±0.0022 | -8±1.19 | 50±5.22 | 72±4.4 |

**
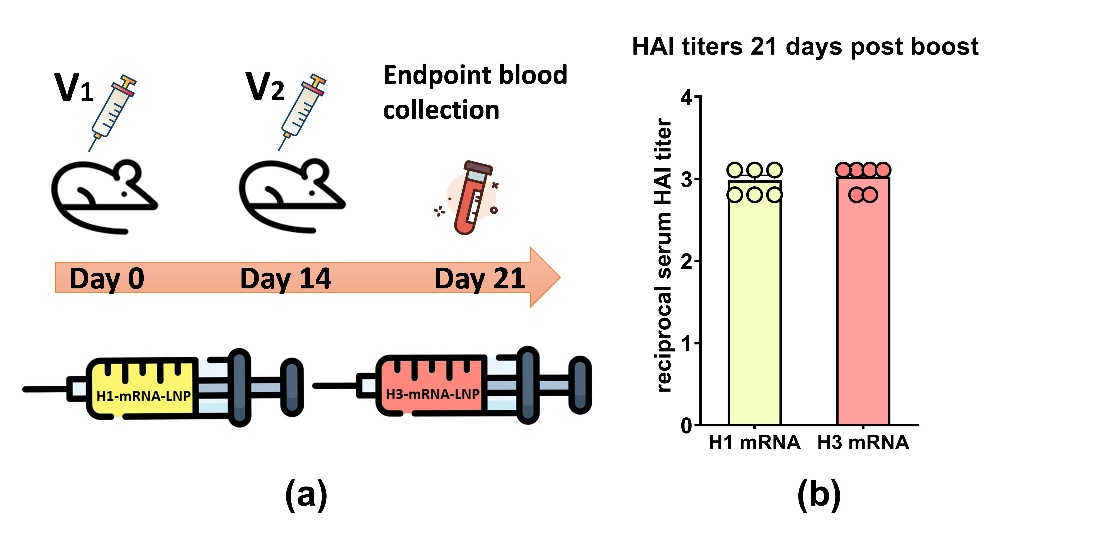
**

**Supplementary Figure 1.** Immunogenicity of H1 and H3 mRNAs mono-vaccines 21 days post second dose. (a) The design of the experiment. Females of BALB/c mice (n=6 in group) were immunized with H1- or H3 mRNA (10 µg per mice) separately. Second doses were administrated 14 days after the primary dose. (b) HAI titers were determined 7 days post second immunization.

**Cross-reactivity of immune response in mice after vaccination with 2.5 µg dose of H1 mRNA mono-vaccine.**

Assuming that over the past four seasons, the most frequent strain changes have occurred in the H1 component of vaccines, we decided to study the cross-reactivity of the immune response against four H1N1 strains after immunization of mice with H1 mRNA (encoding HA A/Wisconsin/588/2019). For this purpose, females of BALB/c mice (n=5 per group) aged 6-7 weeks were immunized twice with an H1-mRNA-LNP (2,5 μg per mouse) with an interval between immunizations of 21 days. The vaccine dose was lower than in experiment with trivalent mRNA vaccine and the time interval between doses was longer by 1 week. The level of immune response was determined by HAI test in the sera of vaccinated mice selected on days 14 and 39 from first vaccination (14 days after prime dose and 18 days after second dose, respectively, Figure 5).

When analyzing immunogenicity in HAI test, antigens of influenza A H1N1 virus strains (A/Wisconsin/588/2019 (clade 6B.1A.5a.2), A/California/07/2009 pdm (clade 6B.1.), A/Guangdong-Maonan/SWL1536/2019 (clade 6B.1A.5a.1), A/Moscow/52/2022 (clade 6B.1A.5a.2a) were used, which belong to different genetic clades of pandemic influenza A H1N1 and have different degrees of homology with the H1 component of the mRNA-IV vaccine by amino acid sequence of HA. Pairwise comparisons of HAI titers to homologous and heterologous antigens revealed a statistically significant decrease in HAI titers to A/California/07/2009 pdm virus antigen in sera on days 14 and 39 after the start of the experiment.


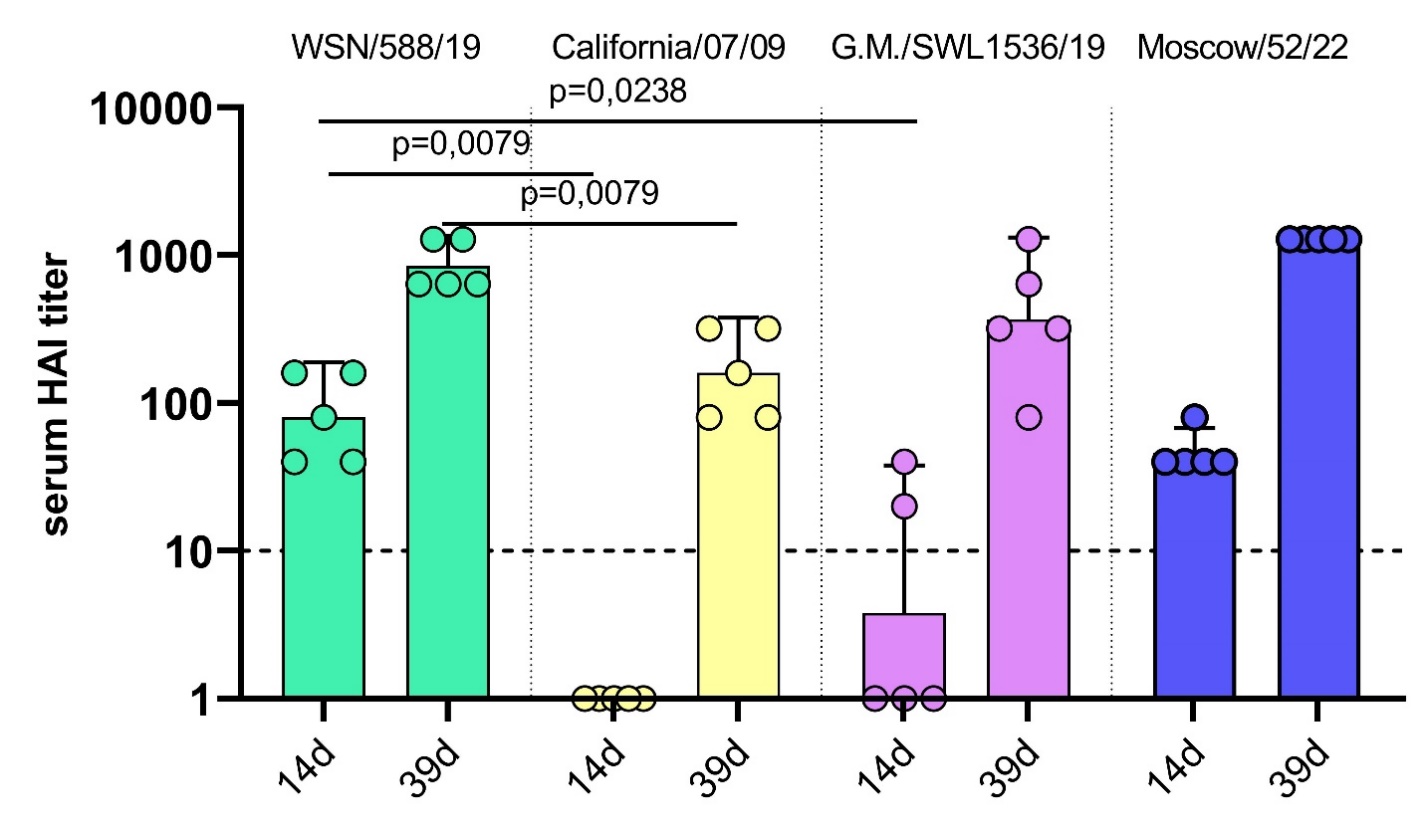


**Supplementary Figure 2.** Cross-specific immunogenicity of 2.5 µg H1-mRNA in mice. HAI titers were determined in serum of mice vaccinated with H1-mRNA (n=5 per group) using flu antigens from four different strains of H1N1 influenza A virus (A/Wisconsin/588/2019 (100% amino acid identity of HA sequences), A/California/07/2009 pdm (95.2% HA identity), A/Guangdong-Maonan/SWL1536/2019 (98.6% HA identity), A/Moscow/52/2022 (98.4% HA identity) at 14 and 39 days after first dose. Data are representative of one experiment and shown as geometric means ± SD. Data were compared using a Mann Whitney test.

The decrease in the mean titer value for A/California/07/2009 pdm on day 39 was 5,2-fold. A less than 4-fold decrease in mean HAI titer (2,2-fold) was observed to A/Guangdong-Maonan/SWL1536/2019 virus antigen on day 39 compared to the homologous (A/Wisconsin/588/2019), with no significant differences (Figure S2). And the mean titer in the serum of mice on day 39 in reaction with the antigen of epidemic strain A/Moscow/52/2022 exceeded that in HAI test with homologous antigen by 0,3 times and the titers to these antigens are not statistically different at both time points (days 14 and 39).


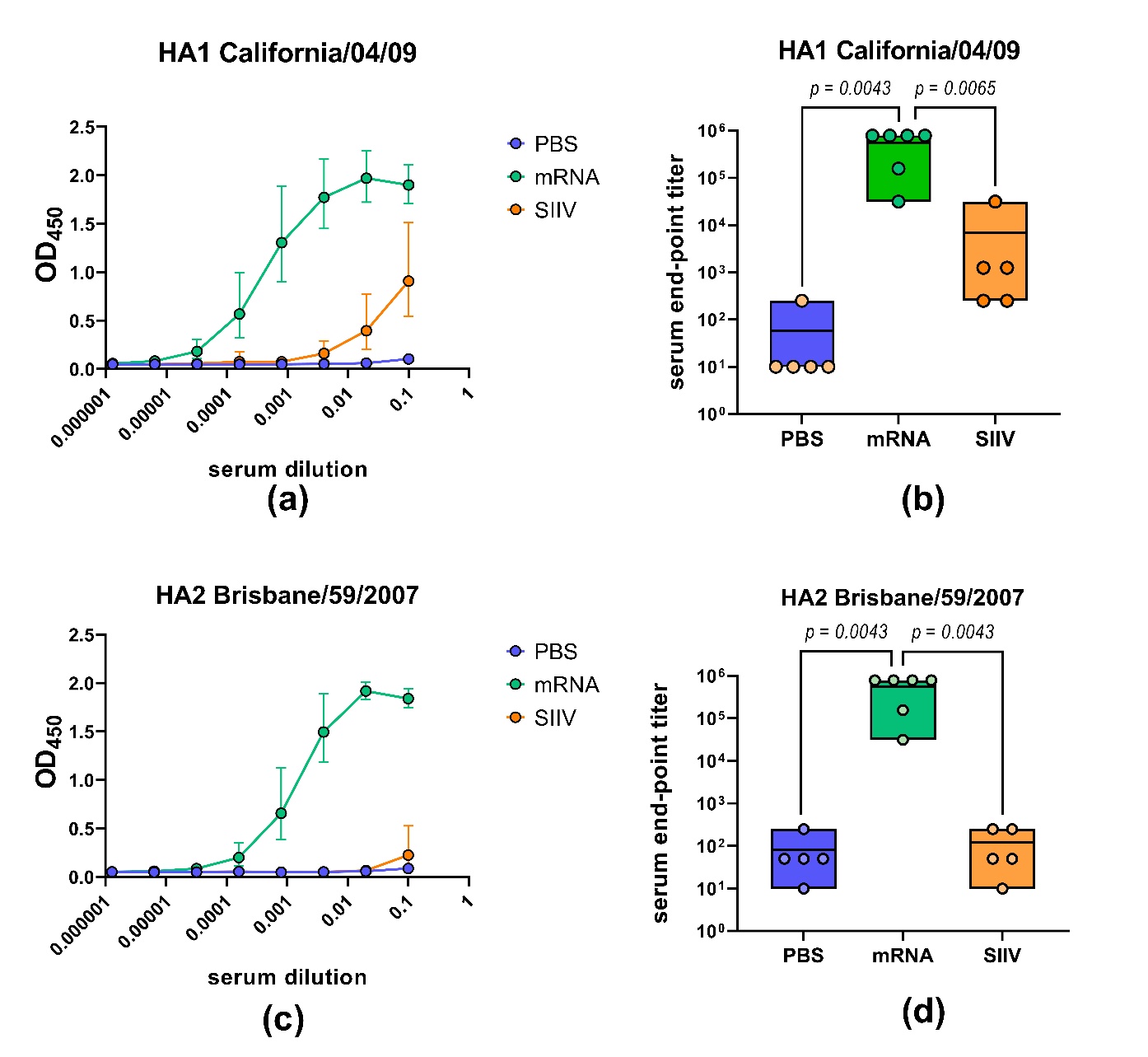


**Supplementary Figure 3.** Serum antibody level to the HA subunits of distant influenza A strains as possible nature of cross-specific immunogenicity of trivalent mRNA vaccine. **(a, c)** The ELISA curves presented with geometric means through group. **(b, d)** End-point titers of mice’s serum in ELISA, determined as highest dilution of serum sample that gives an OD_450_ reading above the cut-off value (mean OD_450_ of negative samples + 3SD).
